# Necrology

**Published:** 1897-06

**Authors:** 


### NECROLOGY.

HERR CARNATENN HARMS, Professor of the Veterinary School of Hanover, author of *A Treatise on Veterinary Obstetrics* and *The Diseases of the Ox and their Treatment,* a graduate of the Hanover School, with additional courses at Berlin and Alfort.

